# Systematic Review of Primary Hyperparathyroidism in India: The Past, Present, and the Future Trends

**DOI:** 10.1155/2011/921814

**Published:** 2011-05-26

**Authors:** P. V. Pradeep, B. Jayashree, Anjali Mishra, S. K. Mishra

**Affiliations:** ^1^Narayana Medical College and Superspeciality Hospitals, Nellore, Andhra Pradesh, India; ^2^Sanjay Gandhi Postgraduate Institute of Medical Sciences, Lucknow, Uttar Pradesh, India

## Abstract

Primary hyperparathyroidism (PHPT) has become an asymptomatic disease in the Western world with the introduction of routine calcium screening. However, the same phenomenon is not observed in India. We have now systematically reviewed the status of PHPT in India. While there is a paucity of literature on PHPT from India when compared to Western countries, some information can be gleaned upon. Most patients present with symptomatic disease whereas very few are screen-detected cases (bone disease 77%, renal disease 36%, and 5.6% asymptomatic). Mean calcium, parathyroid hormone (PTH), and alkaline phosphate levels are high while Vitamin D levels are low. The average parathyroid gland weight is large and the majority being parathyroid adenomas (89.1%). Hungry bone syndrome (HBS) is common in the postoperative period. The disease-related mortality rate is 7.4%, recurrence 4.16%, and persistent disease 2.17%. We suggest that dedicated efforts are needed to pick up asymptomatic disease in India by methods like incorporating calcium estimation in the routine health check-up programs.

## 1. Introduction

Primary hyperparathyroidism (PHPT) is a disease characterized by hypercalcemia due to autonomous production of parathyroid hormone (PTH). PHPT is present in 1% of the adult population, and its incidence increases to 2% after the age of 55 years in Western series [1]. With the advent of multichannel biochemical screening in the 1970s, the incidence of PHPT increased around the world. Subsequently, the clinical entity of asymptomatic hyperparathyroidism was recognized [2]. There are striking discrepancies around the world with respect to incidence, symptoms, and complications of PHPT. In developing countries, particularly India, PHPT is still an uncommonly diagnosed, overtly symptomatic disease of “bones, stones, abdominal groans, and psychic moans.” This may be because of the fact that, in India, screening of the healthy population for hypercalcemia is not a routine practice and there is limited access to medical treatment, especially in the rural areas. Contemporary series of patients with PHPT from developed nations are largely dominated by elderly females with mild to moderate hypercalcemia and very few with classical symptoms [3], contrary to the clinical picture seen in developing countries, especially India. This study was conducted to systematically review PHPT in India both in the past (before the year 2000) and the present (after 2000). We also explored the methods which would possibly change the present trend, so as to diagnose more cases at an asymptomatic stage in order to decrease the morbidity and mortality seen in Indian patients with PHPT.

## 2. Our Findings and Observations

**Figure 1:**
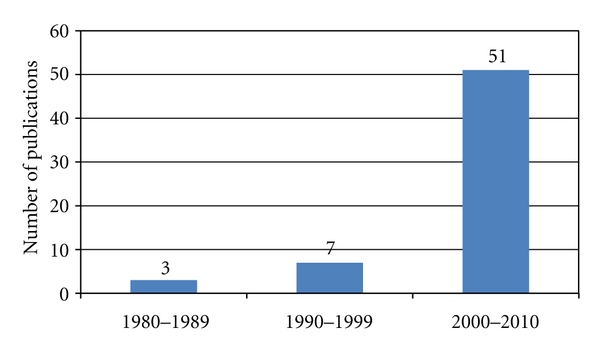
Decade wise publications on PHPT from India.

**Figure 2:**
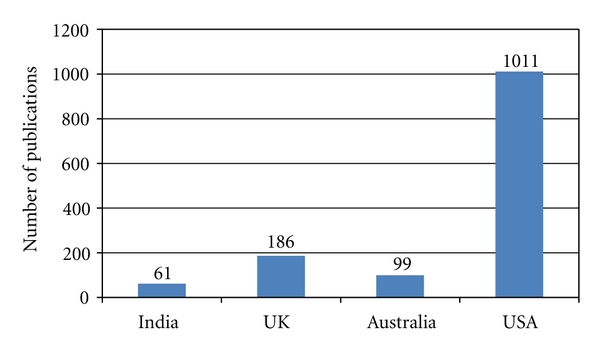
Publications on PHPT: India versus others.

There are few publications on PHPT from India when compared to that of countries like USA, UK, and Australia. The majority of these publications are case reports, small case series, and retrospective case series of approximately 100 cases. These publications lack uniformity in the presentation, analysis, interpretation of data, and the specific information needed for a meta-analysis and appropriate statistical tests. Sixty-one publications related to PHPT from various Indian centers containing data related to 858 PHPT patients were found and included in this study (1980–2010, retrospective reviews, case reports). Publications on PHPT from India are limited to few select centers [4–10]. The majority (twenty-one) of publications were from Sanjay Gandhi Postgraduate Institute of Medical Sciences, Lucknow (SGPGIMS) from which the data of the first 100 cases is included in this study [4, 5].  Figure 1 shows the decade-wise publications from India since the first publication in 1980 [11]. The increase in the number of publications on PHPT between 2000–2010 compared to the previous decade is probably due to the increasing parathyroid awareness among the physicians; however, the number of publications are far less when compared to the developed countries.  Figure 2 shows the comparative number of publications from India and the rest of the world.

## 3. Demographics

In Indian series, females were commonly affected (1.7 : 1, F : M). 71.5% of the cases were less than 40 yrs of age whereas patients from developed nations are diagnosed in the fifth and sixth decades. The mean duration of symptoms was from 84 ± 56.7 months [6, 11–13] which indicate the delay in diagnosis.

## 4. Clinical Presentation

**Figure 3:**
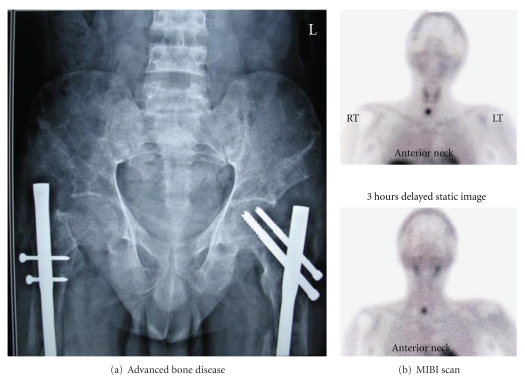
(a) Reveals advanced bone disease with fractures of femur (fixed by rod and nails) and (b) the MIBI scan in a patient with parathyroid adenoma.

**Figure 4:**
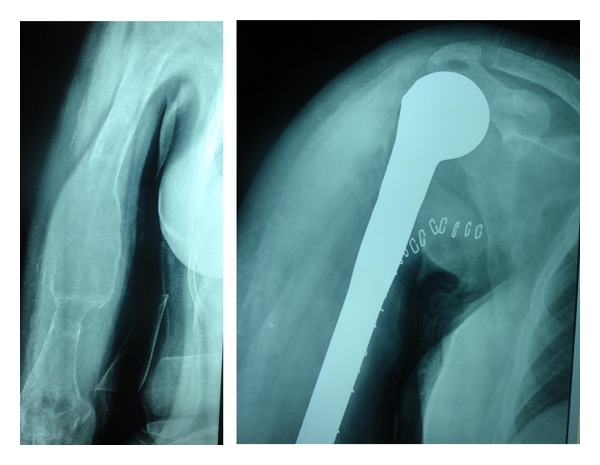
Advanced bone disease (OFC) excised and replaced with humerus prosthesis in a patient with PHPT where the diagnosis was missed.

Worldwide, the presentation of PHPT has changed from a symptomatic to an asymptomatic disease [2]. As a result, newer guidelines are being laid down to decide on indications for surgery in asymptomatic disease [15]. In India, however, asymptomatic presentation is virtually unheard of. Even the symptomatic disease is picked up late after a series of management for fractures and renal stones by the orthopedic surgeons and the urologist.  Figure 3(a) reveals the advanced bone disease in one such patient.  Figure 4 shows a case where a brown tumor was excised and replaced by prosthesis as the diagnosis of PHPT was missed. Data from SGPGIMS, India [4, 5] revealed fractures in 57% of the patients, brown tumors in 49%, and 27% of patients were crippled (due to multiple fractures). Combined bone and kidney disease was seen in 36% of patients, psychiatric symptoms in 38%, and palpable neck masses in 33%. Bhansali et al. [6] have observed similar figures from another premier institute in India where 67% had bone disease, 48% had fractures, 21% had stone disease, 23% had psychiatric symptoms and 15% had peptic ulcer. Among the Indian patients, 5 to 33% had a clinically palpable parathyroid gland. The data we studied revealed that symptomatic disease is seen in all the Indian centers. The overall data related to clinical presentation of Indian PHPT is shown in Table 1 [6, 11–13, 15–26]. The disease spectrum in patients from Pakistan [27], Jordan [28], Turkey [29], Saudi Arabia [30], Hong Kong [31], and Iran [32] are similar. The reasons for higher incidence of osteitis fibrosa cystica (OFC) in Indian patients could be due to Vitamin D (Vit D) deficiency together with the fact that there is a delay in seeking medical attention and, therefore, a delay in diagnosis.

## 5. Biochemical Parameters and Localization Strategies

At SGPGIMS [5], the patients had a mean serum calcium levels of 12.3 ± 1.4 (range 10.8–15 mg %), serum alkaline phosphatase (ALP) 1544.3 ± 2077 IU/L (range 177–7240 IU/L), and serum Vit D levels of 13.6 ± 6.3 (range 6.2–25 ng/mL). Similar biochemical profiles have been reported from other Indian centers (Table 2). Vit D levels in PHPT have been reported only from few studies from India. Priya et al. [7] reported Vit D levels of 10.21 ± 5.82 ng/mL in their series of 39 patients. Pradeep et al. [5] also reported similar low Vit D levels in patients with PHPT (Table 2). Table 2 also shows the serum calcium, PTH, and Vit D levels from Indian centers as compared to a large series of 10,000 cases from the USA. The levels of serum calcium, PTH, and Vit D were significantly different from that of the USA (Table 2). Studies from Hong Kong [34], Israel [35], and South Africa [36] have shown that the introduction of autoanalyzers for calcium detection have enabled them to pick up early disease in older subjects. Lo et al. [34] observed that in Hong Kong with introduction of calcium screening, the age of presentation of PHPT advanced from 45 to 57 years over a three-decade period of study [37]. However, this was not the case resulting in early diagnosis of PHPT in India, in spite of increasing awareness of the disease. Routine health checkups initiated by a majority of corporate hospitals in India do not include estimation of serum calcium. Unlike the developed western nations, the serum calcium, serum parathyroid hormone, and serum alkaline phosphate levels in patients from India have been found to be high (Table 2). A majority of Indian patients also have Vit D deficiency [38], which is similar to reports from countries like Pakistan [27] and Jordan [28]. Overall PHPT has advanced clinical and biochemical features in Indian subjects.

There is a paucity of literature on localization studies in all the Indian publications; however, the reported data are in accordance with the published literature from Western series. Neck ultrasound (USG) has been reported to localize the adenoma in 65–77% of patients [6, 9, 12]. Methyl isobutyl isonitrile (MIBI) scan positivity has been reported in 86.9–100% [12, 17].  Figure 3(b) reveals an MIBI-scan image of a right inferior parathyroid adenoma in one of our patients. Thallium-201 Technetium-99 pertechnetate subtraction has been reported to have a sensitivity of 87–100% [6, 12, 17]. Contrast-enhanced computerized tomography (CECT) of the neck has a sensitivity ranging from 65% to 93.5% in localizing parathyroid adenoma [6, 9, 12]. The reported sensitivity of ultrasound of the neck has been low in India, which could be due to the absence of a dedicated parathyroid sinologist. Therefore, the numbers of cases diagnosed/operated on are far less than what is reported from the developed nations [6, 12, 17, 39]. Even though Thallium-201 Technetium-99 pertechnetate scan and CECT scan of the neck were more commonly used prior to the year 2000 for localization, these are currently replaced with MIBI and USG scan [13, 28, 29, 31].

## 6. Surgical Approach

Limited data is available on the surgical approach to PHPT in most of the literature from India. The approach has ranged from bilateral conventional neck exploration [17] and unilateral neck exploration [5, 12] to focused parathyroidectomy [5]. Focused parathyroidectomy was reported in 38% of cases from one of the centers [5]. Unilateral focused open exploration has been generally performed only in those patients who have concordant USG and MIBI findings. Recurrent PHPT has ranged from zero to 4.16% [5, 6, 11, 13] and persistent PHPT between zero and 2.7% [5, 6, 11–13].

## 7. Pathological Features

The striking difference of PHPT between patients in the developed countries and India is the weight of the parathyroid gland. Study of tumor characteristics in developed countries showed that the average tumor weight was 2–4 gm. Among Indian patients, the tumor weight ranged from 1.25 to 102 gm [6, 9, 11, 18]. In the ten articles which mentioned the histopathology of 366 patients with PHPT, the prevalence of adenoma, hyperplasia, and carcinoma was 89.1%, 6.56%, and 4.37%, respectively, [5, 6, 9, 11–13, 16, 18, 20]. The incidence of parathyroid carcinoma causing PHPT in the various series has been 2.6 to 6% [9, 10, 18]. This is higher than that observed from developed countries (1%) [1].

## 8. Postoperative Course

There is considerable lack of data from India on the postoperative course, morbidity, and mortality. These events have been grossly underreported. Postoperative hungry bone syndrome (HBS) has been observed in 24% to 82% of the cases [5, 6, 9, 12, 18]. Pradeep et al. [5] reported that, out of the 100 consecutive PHPT patients operated on, 79 suffered early symptomatic hypocalcemia and 92 had biochemical hypocalcemia after parathyroidectomy. Maximum fall of serum calcium levels were observed within a mean period of 28 hours (range 12–33). Intravenous calcium gluconate infusion was required for a median period of 6 days (range 3–8 days). Simultaneously, oral calcium carbonate (2 g/day) and calcitriol (1 *μ*g/day) was also needed to control the postoperative hypocalcemia. Associated hypomagnesaemia has to be corrected in some of the patients since it may suppress the function of the residual parathyroid tissue, thereby exacerbating postoperative hypocalcemia [5]. There seems to be a higher incidence of sepsis in patients with severe PHPT undergoing parathyroidectomy, though the cause of this is not yet understood. Postoperative pancreatitis [5, 6, 18] and multiorgan failure are other reported complications. Intravenous calcium infusion used to treat HBS in the postoperative period has resulted in a high incidence of thrombophlebitis. Thus, the immediate postoperative period is turbulent in Indian PHPT patients due to HBS, postoperative pancreatitis [17, 39], sepsis, multiorgan failure, metabolic disturbances related to the end-stage renal disease, and thrombophlebitis related to intravenous calcium infusions. In contrast to this, parathyroidectomy in the Western series is an outpatient procedure with the majority getting discharged the same day.

The reported mortality rates after parathyroidectomy in Indian subjects are as high as 7.4% [5, 6, 11]. 50% of the mortality has been due to end-stage renal disease, 16.6% due to metastasis [40], and others due to sepsis and unknown causes.

## 9. Long-Term Followup

Data on long-term followup of patients from India with advanced PHPT is scarce. Recently, the long-term followup after parathyroidectomy (2–13 yrs) was reported from SGPGIMS [5]. According to this study, ALP normalized within 4 to 18 months and healing of the cortical bones occurred in 2–7 months. At a median followup of 3 years, the gain in bone mineral density (BMD) was 48.99% at the hip, 48.66% at the lumbar spine, and 39.84% at the forearm. This gain was more in the Vit D sufficient group when compared to the patients who were Vit D deficient preoperatively and continued to be so after parathyroidectomy [5]. Similar healing of bone lesions was noticed by Bhansali et al. [6] (mean followup 3.82 ± 2.96 yrs). Among the patients with renal stones, 68.7% did not have any further episodes of renal colic [5]. However, the mortality was more in the patients who had nephrocalcinosis/hydronephrosis. Only one study has assessed the neuropsychiatric manifestations in Indian PHPT and their recovery to date [21]. This study showed significant improvement in the Comprehensive Psychopathological Rating Scale at six weeks after parathyroidectomy and no impairment was noted in memory and intelligence. Anemia and marrow fibrosis improved after curative parathyroidectomy in a series of 28 patients at three months of followup [22]. The incidence of recurrent hyperparathyroidism ranged from zero to 4.16% in different series [5, 6, 11, 13]. Postoperative persistent disease has been noted in up to 2.7% of patients [5, 6, 11–13].

## 10. Summary

In conclusion, a majority of patients diagnosed with PHPT in India have symptomatic disease. The mean calcium, PTH, and alkaline phosphate levels are high with low Vit D levels. Vit D deficiency contributes to the severity of bone disease. HBS in the postoperative period necessitates intravenous calcium supplements and prolonged hospital stay, resulting in higher morbidity.

Patients might benefit from early diagnosis of mild and asymptomatic PHPT only if more efforts are taken to identify these cases. For this to be possible, efforts are needed to incorporate calcium screening in the routine health checkups. The increasing parathyroid awareness among physicians as evidenced by the increase in the number of publications on PHPT will help in diagnosing more cases. Similarly, the introduction of courses (DM and MCh) in endocrinology and endocrine surgery by the Indian Medical Council will increase the awareness among practicing clinicians in the area of endocrinology, which in turn will lead to better diagnosis of PHPT. Awareness among the orthopaedicians, urologists, nephrologists, and rheumatologists in India also has to increase since in India the majority of patients are initially diagnosed by them. We also see a need for organizing continuing medical education programs targeting these specialists and general practitioners, especially by those centers who are managing parathyroid diseases. Until then, patients with PHPT in India will continue to be diagnosed late and present with florid manifestations.

## Figures and Tables

**Table 1:** Clinical characteristics of Indian PHPT and the developed world.

	Total number of subjects for which data was available from India	Percentage affected (India)	Percentage affected (developed countries)*
Bone disease	344	77	5
Fractures	399	40.1	ND
Brown tumors	233	42	3
Renal disease	344	36	15
Proximal muscle weakness	357	54.1	ND
Pancreatitis	302	15	Nil
Psychiatric symptoms	246	26.4	ND
Asymptomatic disease	246	5.6	>80%

*Ref [14].

ND: no data.

**Table 2:** Comparison of biochemical profile in PHPT from various Indian institutes and a center in USA.

	Inst: 1 [4, 5] (*n* = 100)	Inst: 2 [8] (*n* = 88)	Inst: 3 [6] (*n* = 46)	Inst: 4 [7] (*n* = 79)	Inst: 5 (*n* = 15) (unpublished data)	USA [33] (*n* = 10000)	Comparison of Indian versus USA study*
Serum calcium (2.12–2.49 mmol/l)	3.14 ± 0.41 (2.55–4.24)	2.97 ± 0.25	2.8 ± 0.3	3.11 ± 0.44 (2.27–4.04)	3.2 ± .31 (2.4–4.1)	2.71 ± .15	*P* < .001 (with institutes 1, 2, and 4) *P* < .01 (with 3 & 5)
Serum PTH (11–65 pg/mL)	1005.8 ± 760.3 (66–3250)	623 ± 714	885.3 ± 613.2	866.6 ± 639.5 (52–3820)	926.2 ± 712.5	105.8 ± 48 pg/mL	*P* < .001 (with institute 2) *P* < .01 (with 1, 3, 4, &5)
Serum ALP (<150 IU/L)	1466.5 ± 1547.6 (98–7240)	426 ± 549	NA**	762.2 ± 754.8 (50–4930)	789.1 ± 452.3	NA	
Serum Vit D (<20 ng/mL Deficiency)	11.6 ± 8.74 (2–44)	NA	NA	NA	12.5 ± 6.45	22.4 ± 9	*P* < .001 (with institutes 1 & 5)

Indian institutes: institute 1: SGPGIMS, Lucknow; institute 2: CMC Vellore; institute 3: PGIMER Chandigarh; institute 4: KEM Mumbai; institute 5: NMCH Nellore.

*Inferential statistics between groups was performed using one-way ANOVA, followed by Newman-Keuls multiple comparison test.

**NA: Not available.

## Appendix

For the present study, PubMed search for publications on PHPT from India was performed with the key words “hyperparathyroidism, India, and names of the individual centers.” Indian data available on the demographics, clinical presentation, localization, operative approaches, postoperative course, and the long-term followup were analyzed. Information on PHPT was also obtained from the internet, conference proceedings, presentations, and personal communications with other centers in India. A major limitation of this literature review on PHPT from India has been the lack of uniformity of data provided in the different studies. Due to this, the statistical tests could not be applied to a major part of the data. Data has been presented as means ± SD, median, and ranges. Inferential statistics between groups (Table 2) was performed using one-way ANOVA, followed by Newman-Keuls multiple comparison test.
